# Cantilever Sensors

**DOI:** 10.3390/s19092043

**Published:** 2019-05-01

**Authors:** Erwin Peiner, Hutomo Suryo Wasisto

**Affiliations:** 1 Institute of Semiconductor Technology (IHT), Technische Universität Braunschweig, Hans-Sommer-Straße 66, D-38106 Braunschweig, Germany; h.wasisto@tu-braunschweig.de; 2 Laboratory for Emerging Nanometrology (LENA), Technische Universität Braunschweig, Langer Kamp 6a/b, D-38106 Braunschweig, Germany

A cantilever is considered the most basic mechanical spring-mass system and has enormous application potential for sensors. From the collected papers in this special issue, several important sensing fields have been demonstrated, i.e., biosample stiffness measurements [1], surface topography and mechanical properties analysis by fast scanning and contact resonance measurements [2], viscosity–density sensing in liquid media [3], vibration monitoring in remote and harsh environments [4], low-voltage electrostatic activation of resonant cantilever devices [5], atomic force microscopy (AFM) in vacuum [6], and high-sensitive, fast-responding quartz-tuning-force AFM [7]. The rather large cantilevers considered in this special issue, with dimensions typically in the hundreds-of-micron to several-millimeter range, are manufactured using both well-established semiconductor planar-technology-based micromachining [2,3,5,6] as well as unconventional fabrication methods using emerging materials [1,4,7]. Primary physical parameters (e.g., force, acceleration, stiffness, density, and viscosity) to be sensed by quasistatic cantilever deflection [1,2,4] or its operation in a resonant mode [2,3,5,6,7] are converted into cantilever deflection and stress/strain induced in the cantilever spring. Electrical output signals are then generated from these intermediate mechanical parameters by optical [4] or capacitive [5] detection of cantilever bending. Alternatively, stress/strain generated in the cantilever spring can be read out using a piezoresistive strain gauge [2] or by piezoelectric transduction [3,6,7]. 

Biocompatible large cantilevers were designed for long-term stiffness experiments with zebrafish embryos, revealing significant stiffness changes during the growth of the embryos [1]. The cantilevers were cut from spin-coated polydimethylsiloxane (PDMS) and fixed to the arms of a microtweezers as force-sensing tips. Quasistatic cantilever bending and biosample indentation was measured using pattern matching and tracking of optical images. Before, the Young’s modulus of PMDS, which depends on fabrication parameters, was calibrated against a reference cantilever yielding 1.70 ± 0.77 MPa averaged over 10 PDMS cantilevers.

Long, slender piezoresistive silicon cantilevers were used as surface-scanning microprobes for fast measurement of roughness and mechanical properties of high-aspect-ratio microstructures, e.g., inside micro holes of critical flow Venturi nozzles and diesel injection nozzles [2]. After surface scanning at high traverse speeds up to 15 mm/s, wear of integrated silicon tips was observed and measured using a tip-testing artifact. Using a compact scanning setup with an integrated feed-unit, first measurements of the elastic modulus of a polymer surface were reported employing the contact resonance technique, which shall in the future enable simultaneous high-speed roughness and mechanical-parameter measurement. 

Roof-tile-shaped resonant modes of microcantilevers excited by AlN-based piezoelectric actuation electrodes were employed for liquid media monitoring applications, e.g., as viscosity–density sensors [3]. By analyzing a competition between two loss mechanisms of different dependences on geometry and combining suitable dimensions with the order of resonant mode, the *Q*-factor could be maximized. Based on these results, density and viscosity resolutions of 10^−6^ g/mL and 10^−4^ mPa×s, respectively, were estimated for an optimized cantilever geometry corresponding to a resonance frequency less than 1 MHz. 

A high-sensitivity, robust, and easy-to-fabricate cantilever based on a Fabry-Perot interferometer (FPI) with an in-fiber collimator was reported for low-frequency mechanical vibration monitoring in remote and harsh environments [4]. The sensor is composed of a quarter-pitch graded-index fiber spliced with a section of a hollow-core fiber, which is interposed between single mode fibers. With the fiber collimator, a static displacement sensitivity was measured with the FPI of 5.17 × 10^−4^ μm^−1^, and the vibration sensitivity at 100 Hz of the FPI with collimator was reported to be 60.22 mV/g.

Electrostatic actuation of micro cantilever resonators was found to be suffering from high-voltage actuation requirements and high noise low-amplitude signal-outputs, which can be improved via double resonance excitation using a mixed-frequency signal that triggers the mechanical and electrical resonances of the device simultaneously [5]. A coupled mechanical and electrical mathematical linearized model was proposed for different operation frequencies and validated experimentally with a commercial accelerometer based on out-of-plane-displacement of a paddled double-cantilever. Amplification of the voltage across the resonator and the amplitude by factors of 21 and 31, respectively, were reported.

Silicon cantilevers with integrated piezoelectric thin films for a tunable *Q*-factor were reported for operating an AFM cantilever at high speed and high spatial resolution under vacuum conditions, e.g., in a scanning electron microscope [6]. Via the integrated aluminum nitride piezoelectric thin film, a stimulus is generated to the cantilever, which has a phase shift with respect to the excitation by the external piezoelectric actuator underneath the cantilever. A *Q*-factor reduction by a factor of about 1.9 (electrically) and 1.6 (optically) was observed.

Pulled-quartz nanorod cantilevers with an apex diameter of about 150 nm mounted into a frequency modulation (FM)-mode quartz-tuning-force (QTF) AFM system were investigated with respect to their buckling-based nonlinear dynamic mechanical response [7]. Enhanced sensitivity, reduced response time, and a measurement capability of bidirectional mechanical perturbations were found. Using the buckling instability high-sensitive detection of lateral and perpendicular surface acoustic waves was achieved at a bandwidth-limited response time of less than 1 ms.

In summary, cantilever sensors from different materials (i.e., silicon, quartz, PDMS) were described for measuring physical parameters, e.g., force, acceleration, stiffness, density, and viscosity, in various ambient media including laboratory, industrial, and vacuum conditions or in liquids. Both quasistatic and dynamic operation modes were considered with external actuation or excitation using integrated electrostatic electrodes or piezoelectric thin layers. Either vision-based or integrated piezoresistive, piezoelectric, capacitive, or fiber-optical sensing schemes were selected for tactile sensing with biological tissue, machined workpieces, organic layers, and in AFM as well as inertial sensing (vibration, acceleration) and liquid-substance analysis. In this sense, this special issue represents a dedicated survey of advanced cantilever-based methods based on unconventional dimensions, materials, and actuation/sensing schemes for various applications in research and industry.

## References

[1] Tomizawa Y., Dixit K., Daggett D., Hoshino K. (2019). Biocompatible Cantilevers for Mechanical Characterization of Zebrafish Embryos using Image Analysis. Sensors.

[2] Brand U., Xu M., Doering L., Langfahl-Klabes J., Behle H., Bütefisch S., Ahbe T., Peiner E., Völlmeke S., Frank T., Mickan B., Kiselev I., Hauptmannl M., Drexel M. (2019). Long Slender Piezo-Resistive Silicon Microprobes for Fast Measurements of Roughness and Mechanical Properties inside Micro-Holes with Diameters below 100 µm. Sensors.

[3] Ruiz-Díez V., Toledo J., Hernando-García J., Ababneh A., Seidel H., Sánchez-Rojas J.L. (2019). A Geometrical Study on the Roof Tile-Shaped Modes in AlN-Based Piezoelectric Microcantilevers as Viscosity–Density Sensors. Sensors.

[4] Du B., Xu X., He J., Guo K., Huang W., Zhang F., Zhang M., Wang Y. (2019). In-Fiber Collimator-Based Fabry-Perot Interferometer with Enhanced Vibration Sensitivity. Sensors.

[5] Hasan M.H., Alsaleem F., Ramini A. (2019). Voltage and Deflection Amplification via Double Resonance Excitation in a Cantilever Microstructure. Sensors.

[6] Fischeneder M., Oposich M., Schneider M., Schmid U. (2018). Tuneable Q-Factor of MEMS Cantilevers with Integrated Piezoelectric Thin Films. Sensors.

[7] An S., Kim B., Kwon S., Moon G., Lee M., Jhe W. (2018). Buckling-Based Non-Linear Mechanical Sensor. Sensors.

